# Case Report: Torque teno virus identified in pleural effusion of pediatric severe Mycoplasma pneumoniae pneumonia: diagnostic and therapeutic implications

**DOI:** 10.3389/fped.2026.1801533

**Published:** 2026-06-02

**Authors:** Jiaxin Liang, Hanwei Ma, Yuanxiao Li, Xingxing Dai, Xingchuan Li

**Affiliations:** 1 The Second Hospital & Clinical Medical School, Lanzhou University, Lanzhou, China; 2 Department of Pediatric Gastroenterology, Lanzhou University Second Hospital, Lanzhou, Gansu, China; 3 Department of Pediatric Outpatient and Emergency, Lanzhou University Second Hospital, Lanzhou, Gansu, China

**Keywords:** drug-resistant mycoplasma pneumoniae, pleural effusion, severe mycoplasma pneumonia in children, TNGS, TTV

## Abstract

Mycoplasma pneumoniae pneumonia (MPP) is a common cause of community-acquired pneumonia (CAP) in school-aged children, among whom 1%–20% may develop pleural effusion. Most effusions are small in volume and self-resolving. We report two pediatric cases of severe MPP complicated by massive pleural effusion that were unresponsive to initial anti-mycoplasma therapy. Targeted next-generation sequencing (tNGS) of pleural fluid identified macrolide-resistant Mycoplasma pneumoniae and Torque teno virus (TTV) in both cases. Both patients recovered after closed thoracic drainage, adjustment of anti-mycoplasma therapy according to resistance findings, and adjunctive immunomodulatory treatment. These cases indicate that, in severe MPP with massive pleural effusion, pleural-fluid tNGS may provide useful diagnostic information beyond conventional testing, including the identification of macrolide resistance and additional viral findings such as TTV. However, given the high background prevalence of TTV in humans and the absence of control samples in this report, the clinical significance of TTV detection in pleural fluid remains uncertain. At present, TTV is more appropriately interpreted as a possible biomarker of immune perturbation or disease severity rather than a confirmed pathogenic driver. Broader pathogen testing in selected specimens such as pleural fluid may complement diagnostic evaluation in complex infections, but larger controlled studies are needed to clarify the significance of TTV in this setting.

## 1 Introduction

Mycoplasmal pneumonia (MPP) is one of the most common types of community acquired pneumonia (CAP) in children aged 5 years and older in China. Clinically, approximately 1%–20% of children with MPP may develop pleural effusion, but in most cases, the effusion is small and free-flowing, and can be spontaneously absorbed as the pneumonia improves (1). When a single lung lobe is affected over an area of two-thirds or more, or when two or more lobes present with high-density consolidation accompanied by moderate to large pleural effusion, the condition can be diagnosed as severe pneumonia (2). Due to the complexity of the disease and the increased difficulty of treatment, such cases require heightened clinical attention. This article reports two clinically distinctive cases: both children met the diagnostic criteria for severe MPP and had large pleural effusions. Initial treatment with standard anti-mycoplasma therapy was ineffective, suggesting the possible presence of unidentified pathogenic factors or pathogens. Subsequently, the pleural effusion samples from the pediatric patients were further subjected to tNGS testing for pathogenic microorganisms. In addition to the detection of drug-resistant Mycoplasma pneumoniae, TTV was simultaneously identified in both cases. Following closed thoracic drainage combined with standard-of-care treatment including appropriate antibiotics and supportive therapy, the children eventually recovered. This article not only aims to enhance clinicians' awareness and understanding of severe mycoplasma pneumonia consolidated with massive pleural effusion in pediatric patients, but also attempts to provide important insights for clinical practice: for such complex infectious diseases with poor response to conventional treatment, attention to the potential presence of unconventional pathogens such as TTV and the implementation of targeted pathogen testing may offer new perspectives and directions for optimizing treatment schemes and achieving precision therapy.

## 2 Case reports

### 2.1 Case 1

**Figure 1:**
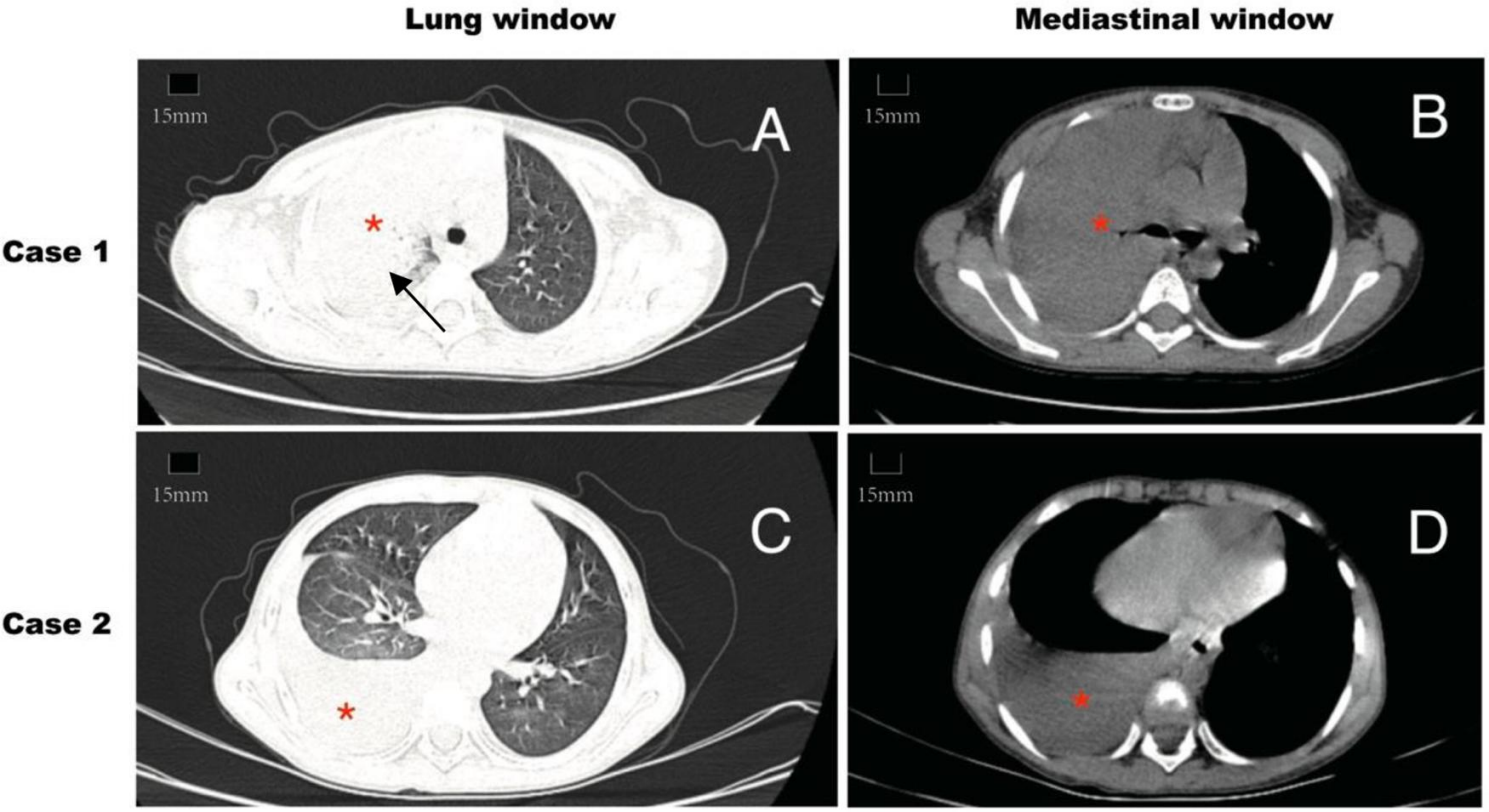
Chest CT manifestations of the two patients. **(A)** Case 1, lung window: right upper lobe bronchial obstruction (→) and consolidation. **(B)** Case 1, mediastinal window: massive right pleural effusion (*). **(C)** Case 2, lung window: right lower lobe consolidation. **(D)** Case 2, mediastinal window: massive right pleural effusion (*). Scale bars = 15 mm.

A 6-year-old boy was admitted with “recurrent fever for 1 week and cough for 4 days.” He presented with persistent high fever (peak 40.5℃, remittent pattern) and worsening dry cough. Chest CT revealed lobar pneumonia in the right upper lobe. After 4 days of intravenous azithromycin, his condition worsened with tachypnea, prompting transfer. Physical examination showed pharyngeal congestion, coarse breath sounds, and diminished breath sounds over the right lung. Laboratory findings: WBC 13.48 × 10^9^/L, NE# 12.23 × 10^9^/L (90.7%), CRP 76.75 mg/L, SAA >320 mg/L, PCT 0.924 ng/mL, IL-6 76 pg/mL, ESR 54 mm/h. Sputum PCR confirmed Mycoplasma pneumoniae. Repeat CT showed right upper lobe bronchial obstruction, lobar pneumonia, and massive right pleural effusion (Figures 1A,B. Ultrasound revealed bilateral pleural effusion (left: 0.43 cm; right: 5.5 cm). Closed thoracic drainage yielded 400 mL of pale-yellow pleural fluid. Pleural fluid analysis: Rivalta test (+), WBC-BF 3,076 × 10^6^/L, TC-BF 3,105 × 10^6^/L (MN# 2,524 × 10^6^/L, PMN# 552 × 10^6^/L). Biochemical analysis: TP 39.4 g/L, ALB 22.5 g/L, ADA 55 U/L, GLU 7.06 mmol/L, LDH 4,306 U/L. NGS detected macrolide-resistant Mycoplasma pneumoniae (7 × 10² copies/mL, 23S rRNA 2063A mutation) and TTV (1 × 10⁴ copies/mL). Sputum culture for common bacteria and acid-fast staining for Mycobacterium tuberculosis were negative. Treatment was adjusted to intravenous levofloxacin for 9 days, IVIG (0.4 g/kg/day ×5 days), and two bronchoscopic lavage procedures. The patient recovered fully, with normal follow-up CT at 1 month.

### 2.2 Case 2

**Figure 2:**
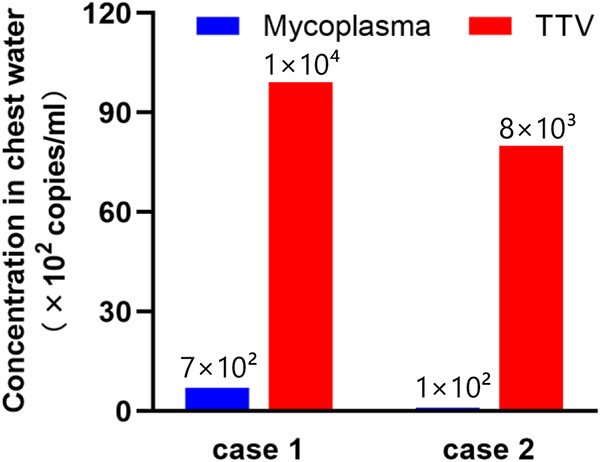
Concentrations of macrolide-resistant Mycoplasma pneumoniae (blue bars) and torque teno virus (TTV, red bars) in pleural fluid samples from two pediatric patients with severe MPP. Quantification was performed by targeted next-generation sequencing (tNGS) using internal standard curves (HugoBiotech). Case 1: M. pneumoniae 7 × 10^2^ copies/mL, TTV 1 × 10^4^ copies/mL. Case 2: M. pneumoniae 1 × 10^2^ copies/mL, TTV 8 × 10^3^ copies/mL. Bars represent single measurements; therefore error bars are not shown. Numerical values are indicated above each bar. *Y*-axis: copies/mL (×10^2^ multiplier).

A 6-year-old boy presented with “intermittent fever for 9 days and cough for 6 days.” He had recurrent high fever (peak 39.8℃, remittent pattern), productive cough, and mild dyspnea. Initial CT showed massive right pleural effusion (Figures 1C,D). Despite azithromycin, penicillin, methylprednisolone, and levofloxacin therapy, symptoms persisted. On admission, physical examination revealed pharyngeal congestion, coarse breath sounds, right-sided diminished breath sounds, and wet rales. Laboratory findings: WBC 15.84 × 10^9^/L, NE# 13.33 × 10^9^/L (84.2%), CRP 35.69 mg/L, SAA >320 mg/L, PCT 0.191 ng/mL, ESR 28 mm/h. Ultrasound confirmed right pleural effusion (7.5 cm) (Figure 2). Closed drainage yielded 430 mL of pale-yellow fluid. Pleural fluid analysis: Rivalta test (+), WBC-BF 1,057 × 10^6^/L, RBC-BF 28 × 10^6^/L, TC-BF 1,150 × 10^6^/L (MN# 967 × 10^6^/L). Biochemical analysis: TP 40.5 g/L, ALB 24.8 g/L, ADA 40 U/L, GLU 6.82 mmol/L, LDH 2,675 U/L. NGS identified macrolide-resistant Mycoplasma pneumoniae (1 × 10² copies/mL, 23S rRNA 2063A mutation) and TTV (8 × 10³ copies/mL). Sputum culture for common bacteria and acid-fast staining for Mycobacterium tuberculosis were negative.Treatment included methylprednisolone (1 mg/kg), doxycycline for 8 days, IVIG (0.4 g/kg/day ×5 days), and two bronchoscopic lavages. The patient recovered fully, with normal follow-up CT at 3 weeks.

## 3 Methods

Clinical specimens were sent to HugoBiotech Co., Ltd. (Shanghai, China) for pathogenic targeted next-generation sequencing (tNGS) detection (DRseq, HugoBiotech, Beijing, China). Briefly, 200 μL of pleural fluid was used to extract DNA and RNA using the Nucleic Acid Extraction Reagent Kit (YGZZ017, HugoBiotech). RNA was reverse-transcribed using the 1st Strand cDNA Synthesis Kit (YG-048, HugoBiotech). Nucleic acids were enriched using the Multi-Pathogen Detection Reagent Kit (YG-032, HugoBiotech), developed based on the Multiprime primer design platform (3).Library construction was performed with the Universal Sequencing Reaction Kit (YGZZ018, HugoBiotech), followed by quality control using Qubit 4.0 (Thermo Fisher Scientific, USA) and Agilent A2100 Bioanalyzer (Agilent Technologies, USA).

Sequencing was conducted on the Illumina 550DX platform (single-end 75 bp). Each batch included a no-template control (NTC) using sterile water. Raw sequencing data were converted from “bcl” to fastq format using bcl2fastq (v2.20.0.422), filtered for adapters and low-quality reads using fastp (v0.24.0) and aligned to the human genome (GRCh38.101) using BWA (v0.7.15) to remove host sequences. Remaining reads were aligned to a local microbial database compiled from NCBI RefSeq and GenBank, and microbial identification was performed based on alignment results.

**Note:** tNGS was performed according to HugoBiotech's proprietary,validated protocols.Detailed metrics such as sequencing depth, copy number conversion, and internal calibration procedures were not provided by the company.

## 4 Discussion

Torque Teno virus (TTV) is a small, non-enveloped, single-stranded DNA virus that is widely distributed in the human population. Since its first identification in 1997, TTV has been detected in approximately 70%–90% of healthy individuals, and its clinical significance remains controversial (4, 5). Increasing evidence suggests that TTV behaves primarily as a commensal virus, and its viral load may reflect host immune status rather than direct pathogenicity.

In the present study, targeted next-generation sequencing (tNGS) identified both macrolide-resistant Mycoplasma pneumoniae and relatively high loads of TTV in pleural effusion samples from two pediatric patients with severe MPP. Both patients improved following standard management, including appropriate anti-mycoplasma therapy, pleural drainage, and immunomodulatory treatment. Notably, no specific antiviral therapy targeting TTV was administered. Therefore, the clinical recovery observed in these cases is more likely attributable to effective treatment of Mycoplasma pneumoniae infection rather than a direct intervention against TTV.

The detection of TTV in pleural effusion, a relatively sterile body fluid, is nevertheless noteworthy. Compared with upper respiratory tract specimens, pleural fluid is less susceptible to contamination and may better reflect local pathological processes (13). The presence of TTV in this compartment may indicate viral reactivation or redistribution under conditions of severe inflammation or immune dysregulation. Previous studies have demonstrated that TTV load is associated with immune status, particularly in immunocompromised individuals, where elevated viral loads are frequently observed (5).

Several studies have explored potential interactions between TTV and the host immune system. TTV has been reported to modulate immune responses through interactions with inflammasomes and other intracellular signaling pathways, thereby influencing immune homeostasis (14). In addition, TTV may affect T-cell function via the HLA-E/NKG2A axis, contributing to immune exhaustion or altered immune regulation (15). In pediatric populations, TTV has also been associated with impaired epithelial barrier function and increased susceptibility to respiratory infections (16). However, these findings are primarily based on experimental or observational studies and do not establish a causal role for TTV in disease pathogenesis.

In the context of the present cases, it is more plausible that elevated TTV levels reflect underlying immune dysregulation associated with severe MPP rather than acting as a primary pathogenic factor. The observed temporal association between higher TTV loads and severe clinical manifestations suggests that TTV may serve as a potential biomarker of disease severity or immune status, rather than a direct driver of disease progression.

Furthermore, alternative causes of pleural effusion were carefully considered. Bacterial co-infection, tuberculosis, viral pleuritis, and immune-mediated mechanisms were evaluated based on clinical presentation, laboratory findings, and treatment response. The available evidence supported a diagnosis of severe MPP with parapneumonic effusion, rather than other etiologies.

Overall, this study highlights the potential clinical value of expanded pathogen detection using tNGS in complex infectious diseases (6–12). However, the interpretation of unconventional viral findings such as TTV should be approached with caution. While TTV detection may provide additional insights into host immune status, current evidence does not support its role as a direct pathogenic factor in severe MPP. Further studies with larger sample sizes, appropriate controls, and mechanistic investigations are required to clarify the clinical significance of TTV in respiratory infections.

## 5 Limitations

This study has several important limitations. First, the sample size is extremely small (*n* = 2), which precludes any causal inference between TTV detection and disease severity. Second, no control group was included, and therefore it is unclear whether the detected TTV levels are higher than baseline levels observed in healthy individuals or in patients with uncomplicated pneumonia. Third, the study lacks longitudinal monitoring of TTV viral load, making assessment of its dynamic relationship with disease progression and treatment response difficult. Finally, mechanistic evidence supporting a pathogenic role of TTV in respiratory disease is lacking, and the observed association may reflect immune dysregulation rather than direct viral pathogenicity.

## Data Availability

The datasets presented in this article are not readily available due to commercial confidentiality restrictions imposed by the sequencing service provider (HugoBiotech), the raw sequencing data are not publicly available. The processed data supporting the findings of this study are presented within the article. The raw data may be made available by the corresponding author upon reasonable request, subject to the approval of the service provider to protect their intellectual property.
